# Effect of ambient storage on the quality characteristics of aerobically packaged fish curls incorporated with different flours

**DOI:** 10.1186/2193-1801-3-106

**Published:** 2014-02-21

**Authors:** Waseem Hussain Raja, Sunil Kumar, Zuhaib Fayaz Bhat, Pavan Kumar

**Affiliations:** Mirah Exports Pvt Ltd, Darabassi, SAS Nagar, Punjab India; Division of Livestock Products Technology, Faculty of Veterinary Sciences and Animal Husbandry, Sher-e-Kashmir University of Agricultural Sciences and Technology of Jammu, R S Pura, Jammu, Jammu and Kashmir 181102 India; Division of Livestock Products Technology, GADVASU, Ludhiana, Punjab 141004 India

**Keywords:** Snacks, Curls, Fish, Flours, Ambient storage, Physicochemical parameters, Sensory attributes

## Abstract

The present study was conducted to evaluate the effect of ambient storage on the quality attributes of aerobically packaged fish curls incorporated with optimum levels of different flours. The curls were developed by extrusion technology using fish meat (*Catla catla*). The fish curls containing optimum levels of different flours viz. 20 percent corn flour, 10 percent black gram flour and 10 percent peanut flour were compared with the control snacks containing 30 percent rice flour and assessed for storage quality and shelf life at ambient temperature. The curls were aerobically packaged in LDPE (low density polyethylene) pouches and evaluated for various physicochemical, microbiological and sensory parameters. Mean values of pH of all the curls showed significantly (p < 0.05) decreasing trend with increasing days of storage (6.34 ± 0.01 on day 0 and 5.90 ± 0.005 on day 28 for control samples, 6.41 ± 0.009 on day 0 and 6.11 ± 0.02 on day 28 for corn flour incorporated samples, 6.36 ± 0.01 on day 0 and 6.14 ± 0.01 on day 28 for black gram flour incorporated samples, 6.57 ± 0.007 on day 0 and 6.34 ± 0.01 on day 28 for peanut flour incorporated samples). TBARS (mg malonaldehyde/kg), total plate count (log cfu/g) and yeast and mould count (log cfu/g) for the control as well as treatment samples showed significantly (p < 0.05) increasing trend with storage. Coliform counts (log cfu/g) were not detected until day 28 in all the products. The mean scores of sensory parameters i.e. appearance and colour, flavor, crispiness, texture and overall acceptability for control as well as treatment samples showed significantly (p < 0.05) decreasing trend with storage period. The decrease was significantly (p < 0.05) highest on 21^st^ and 28^th^ day of storage. The mean values for all the quality and storage parameters up to the day 21 of the storage were within the acceptable limits. Thus, based on various physicochemical and sensory parameters, the curls incorporated with optimum level of different flours were acceptable up to 21 days of ambient storage within the LDPE pouches.

## Introduction

Snacking can be defined as problem free consumption of easy to handle, miniature portioned, hot or cold products in solid or liquid form which need little or no preparation and are intended to satisfy the occasional pangs of hunger (Kumar et al. 2012). Snack food is one of the fastest growing segments of the food industry. Over the decades the consumption of snack food has increased significantly (Thakur and Saxena 2000) and has become integral part of the diet of the world’s population due to several reasons like rapid urbanization, changing life style, increase in number of nuclear families and working women, media penetration, and higher disposable incomes. It is the food of choice for school going children, adolescent girls and high mobility groups. The market of snack food industry including semi-processed/cooked and ready to eat foods was around Rs 82.9 billion in 2004 to 2005 and is rising rapidly with a growth rate of 20% (Singh et al. 2011). Basic criteria for snack foods are convenience, manageable portions and satisfaction of short term hunger (Tettweiler 1991).

Snack products include various types of the products such as cookies, biscuits, pies, sticks, breads, curls etc. Most of the snacks available in the market are mainly based on cereals which are high in calorie and low in protein content. If they are taken in large quantity, they can suppress the appetite for the main meal. For this reason, snack with high protein and high fiber should be developed as a supplementary diet (Prabhavat et al. 2000). Curls are ready to eat quick snacks with several attractive features including wider consumption base, relatively longer shelf-life, more convenience and good eating quality. Curls, like most of the snacks, are mainly prepared from cereal grains and these cereals based snack products lack some essential amino acids like tryptophan, threonine and lysine (Kumar et al. 2012). However, protein content and nutritional value of curls can be increased by the addition of high quality protein sources such as fish, legumes and corn. Thus, the incorporation of fish meat along with legumes and corn in the curls is a good alteration in its nutritional value.

Meat based snacks are important snack foods available in the world market particularly in south-east Asia. Fish based snack foods which are commonly known as fish-crackers are popularly consumed in such countries (Suknark et al. 1998). Fish-crackers are traditionally produced by gelatinization of starch in dough made from flour and fish which is shaped, steamed, cooled, sliced, dried, and packaged for sale in polyethylene bags (Siaw et al. 1985). The chemical composition of the fish is valuable in developing high protein snack foods, while ensuring the finest quality flavor, colour, odour, texture. Many of the snacks particularly meat based are produced by extrusion technology. Extrusion is the art or process of shaping by forcing through the die. It’s used for various processes like cooking, expansion, texture alteration, mixing and utilization of various ingredients. By extrusion technology cereals, tubers, and their derivatives can be transformed into snack foods and deboned meat can be firmly compressed and reshaped into sticks and links. Historically extrusion cooking of 19^th^ century was used to shape sausage products. Extrusion technology has been widely used for the manufacture of meat and flour based snacks by blending meat with various non-meat products like flours (Prinyawinatkul et al. 2007; Shaviklo et al. 2011).

Fish has received increased attention as a potential source of animal protein and essential nutrients for human diets. It is a good source of essential amino acids, fat-soluble vitamins and several polyunsaturated fatty acids such as linoleic acid, eicosapentaenoic acid (EPA) and docosahexaenoic acid (DHA). Thus, fish based curls can prove to be valuable items to the consumer as a source of essential amino acids and other nutrients. Use of fish meat in the development of curls will open novel opportunity for profitable utilization of fish and improve the nutritional aspect of people as well as prosperity of fish industry. Fish meat based snack food is rarely available in most of the markets of India and information on fish curls is more or less non-existent in literature. Furthermore, rapid expansion of fast food market has increased the development of meat products and warrants immediate attention regarding the development of novel meat products like fish curls. Use of fish may also reduce the cost of meat products since the rates of fish in most of the Indian markets, by and large, are lower than other meats.

Legumes are good source of protein and provide energy, protein, minerals, vitamins and dietary fiber required for human health. Inclusion of legumes in the diet has many physiological effects in controlling and preventing various metabolic diseases such as coronary heart disease and colon cancer (Tharanathan and Mahadevamma 2003). Legume flours have widely been used as extenders in meat products in order to reduce the cost of production and improving the nutrition. Partial replacement of meat by cowpea and peanut (Prinyawiwatkul et al. 1997), cowpea, black bean and green gram (Bhat et al. 2011, 2013a), corn flour (Serdarouglu and Degirmencioglu 2004) and black gram (Modi et al. 2003) have been successfully reported. The use of certain legume proteins such as peanut flour and black gram in the formulation of fish curls would improve the nutritional characteristics and may provide superior functional properties to the product.

The present study was aimed at developing the curls using fish meat and protein rich flours using extrusion technology and further evaluating the shelf life of the developed products. Prospects of further developing certain novel meat products like curls by using fish meat and by extending the same with certain flours could find increasing popularity in food service industry particularly at fast food outlets. Thus, the present study was envisaged to evaluate the effect of ambient storage on the quality characteristics of fish curls incorporated with optimum levels of different flours.

## Material and methods

### Fish meat

Fish (*Catla catla*, Indian major carp) were purchased from local market of Jammu. The body scales were removed and eviscerated. Deboning of dressed fish was done manually. The lean meat was packed in polythene bags and frozen at -18 ± 2°C until use.

### Condiment mixture

Condiments used were onion, garlic and ginger. The external covering of all were peeled off and cut into pieces. The cut pieces were weighed in a ratio of 3:2:1 and ground in a mixer to the consistency of fine paste.

### Spice mixture

The spice mix formula used for preparation of the fish curls contained anise (*Pimpinalla anisum*, *soanf*-13%), bay leaves (*Laurus nobilis, tej patta*-2%), black pepper (*Piper nigrum*, *kali mirch*-5%), green cardamom (*Elettaria cardamomum*, choti elaichi-5%), cinnamon (*Cinnamomum zeylanicum, dalchini*-6%), cloves (*Syzygium aromaticum, laung*-2%), dry fenugreek powder (*Foenum-graecum, meathi*-6%), coriander (*Coriandrum sativum, dhania*-20%), cumin seed (*Cuminum cyminum, jeera*-12%), mace (*Myristica fragrans, javitri*-2%), nutmeg (*Myristica fragrans, jaiphal*-2%), red chilli (*Capsicum frutescens, lal mirch*-12%), black cardamom (*Amomum subulatum, badi elaichi*-5%), mint leaves (*Lamiaceae*, *pudina*-3%) and dry ginger powder (*Zingiber officinale*, *saunth*-5%). The spices were purchased from local market. After removal of extraneous matter, all spices were dried in an oven at 50°C for overnight and then ground in grinder to powder. The coarse particles were removed using a sieve (100 mesh) and the fine powdered spices were mixed in required proportion to obtain spice mixture for fish curls. The spice mixture was stored in plastic airtight container for subsequent use.

### Fat

Refined cottonseed oil of brand name ‘*Ginni*’ (Amrit Banaspati Company Limited, India) was purchased from local market and used in emulsion preparation as well as for deep fat frying of the curls. It approximately contained 900 k.cal of energy, 0 g of carbohydrate, 0 g of proteins, 0 g of cholesterol, 24 g of saturated fatty acids, 54 g of mono-unsaturated fatty acids and 0 g of trans-fatty acids per 100 grams.

The fish curls were prepared by incorporating two fat levels viz. 0 percent and 5 percent in the emulsion. The various sensory attributes viz. colour and appearance, flavour, crispness, texture and overall acceptability were significantly (p < 0.05) higher in the curls made from formulation containing 0 percent refined cottonseed oil. Thus 0 percent oil was optimized as best for the emulsion preparation.

### Flours

Refined wheat flour, rice flour and defatted corn flour were brought from local market. Peanuts and black gram were purchased from local market and processed to flour in the division of Livestock Products Technology, SKUAST of Jammu.

On the basis of preliminary trials, incorporation of 30 percent level of rice flour was optimized as best for the development of control fish curls. Rice flour was incorporated at 25 and 30 percent level in the basic formulation of fish curls. The mean values of sensory scores viz. colour and appearance, crispiness, texture and overall acceptability at 30 percent incorporation of rice flour were significantly (p < 0.05) higher as compared to 25 percent rice flour.

The three flours viz. corn flour (approximately containing moisture 2.7%, crude protein 0.40%, crude fat 0%, carbohydrates 96.7%), black gram flour (approximately moisture 10%, crude protein 22%, crude fat 6%, carbohydrates 57%) and peanut flour (moisture 6%, crude protein 27%, crude fat 47%, carbohydrates 19%) were incorporated at three different levels viz. 10, 20 and 30 percent to replace corresponding amounts of rice flour in the formulation of fish curls. Based on various physicochemical and sensory parameters, incorporation of 20 percent corn flour, 10 percent black gram flour and 10 percent peanut flour were optimized as best.

### Preparation of emulsion

**Figure 1:**
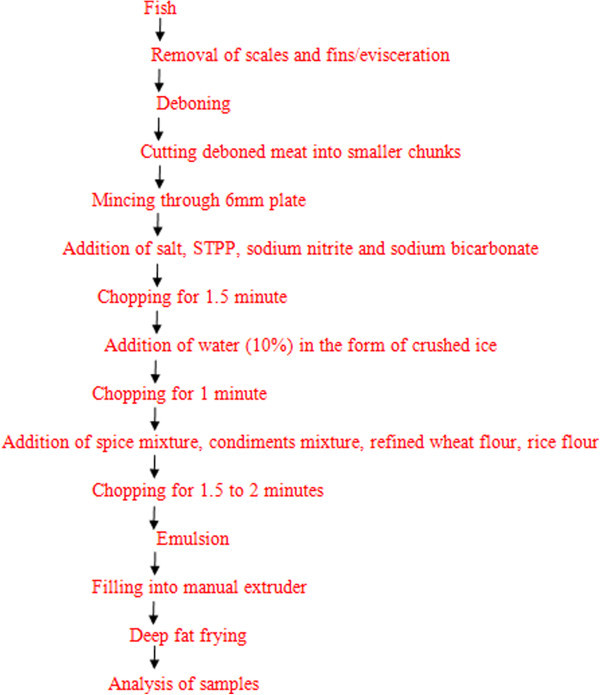
**Flow diagram for the preparation of fish curls.**

Several preliminary trials were conducted to standardize the formulation for preparation of emulsion for fish curls as indicated in Table 1. Figure 1 shows the flow diagram for the preparation of fish curls. Fish meat was cut into smaller chunks and minced in a Sirman mincer (MOD-TC 32 R10 U.P. INOX, Marsango, Italy) with 6 mm plate. Meat emulsion for fish was prepared in Sirman Bowl Chopper [MOD C 15 2.8G 4.0 HP, Marsango, Italy]. Minced meat was chopped with all the curing ingredients (sodium bicarbonate, sodium nitrite, sodium tripolyphosphate (STPP), common salt) for 1.5 minute. Water in the form of crushed ice was added and blending continued for 1 minute. This was followed by addition of spice mixture, condiments and other ingredients and again mixed for 1.5 to 2 minutes to get the desired emulsion. Adequate care was taken to keep the end point temperature below 18°C by preparing the emulsion in cool hours of morning, by addition of meat and other ingredients in chilled/partially thawed form and by addition of crushed ice.

**Table 1:** **Formulation of fish curls with rice, corn, black gram and peanut flour**

Ingredients	RF (30%)	CF (20%)	BGF (10%)	PNF (10%)
Fish meat	47	47	47	47
Added water	10	10	10	10
Condiment mixture	2.7	2.7	2.7	2.7
Rice flour	30	10	20	20
Corn flour	-	20	-	-
Black gram flour	-	-	10	-
Peanut flour	-	-	-	10
Refined wheat flour	5	5	5	5
Spice mixture	2	2	2	2
Table salt	2.5	2.5	2.5	2.5
Sodium tripolyphosphate	0.3	0.3	0.3	0.3
Sodium nitrite	150 ppm	150 ppm	150 ppm	150 ppm
Sodium bicarbonate	0.5	0.5	0.5	0.5

RC = Rice flour (control), CF = Corn flour, BGF = Black gram flour, PNF = peanut flour.

Deep fat frying method was employed for cooking of curls. Emulsion obtained was filled into the manual extruder and curls obtained from the extruder were directly deep fat fried in the refined cottonseed oil. The products were cooked at different time-temperature combinations and based on mean values of various sensory parameters fish curls cooked at 180 ± 5°C for 3 ± 1 minutes were optimized as best. The temperature of the oil was measured by a digital thermometer.

### Analytical procedures

The pH of cooked fish curls was determined by the method of Keller et al. (1974) using a digital meter (Systronics Digital pH Meter 803, serial No. 603). Thiobarbituric acid reacting substances value of fish curls during storage was determined using the method described by Witte et al. (1970). Microbiological profile viz. total plate count, coliform count and yeast and mold count were determined by methods described by APHA (1984). Readymade media (Hi-Media) were used for the analysis.

### Sensory evaluation

The sensory evaluation of the products was carried for various attributes namely colour and appearance, flavour, crispiness, texture and overall acceptability by a panel of seven trained members composed of scientists and research scholars of the Division based on a 8-point hedonic scale, wherein 8 denoted “extremely desirable” and 1 denoted “extremely undesirable” (Seman et al. 1987). Panelists were seated in a room free of noise and odours and suitably illuminated. Coded samples for sensory evaluation were prepared and served warm to panelists. Water was provided for oral rinsing between the samples.

### Statistical analysis

Means and standard errors were calculated for different parameters. Factorial design of experiment was fallowed. Analysis of variance was performed as per Snedecor and Cochran (1994). In significant effects, least significant differences were calculated at appropriate level of significance for a pair wise comparison of treatment means.

## Results and discussion

### Chemical measurements

The mean values of chemical measurements namely pH and thiobarbituric acid reacting substances value of aerobically packaged fish curls containing different flours are presented in Table 2.

**Table 2:** **Effect of ambient storage on the chemical measurements of aerobically packaged fish curls incorporated with rice, corn, black gram and peanut flour (Mean ± SE)** ^*^

Treatment	Storage period (days)				
	0	7	14	21	28
**pH**					
**RF (30%)**	6.34 ± 0.01^Ae^	6.19 ± 0.007^Ad^	6.14 ± 0.01^Ac^	6.07 ± 0.006^Ab^	5.90 ± 0.005^Aa^
**CF (20%)**	6.41 ± 0.009^Be^	6.34 ± 0.007^Bd^	6.28 ± 0.006^Bc^	6.23 ± 0.006^Bb^	6.11 ± 0.02^Ba^
**BGF (10%)**	6.36 ± 0.01^Ac^	6.27 ± 0.006^Cb^	6.25 ± 0.006^Cb^	6.16 ± 0.01^Ca^	6.14 ± 0.01^Ca^
**PNF (10%)**	6.57 ± 0.007^Ce^	6.50 ± 0.005^Dd^	6.44 ± 0.008^Dc^	6.39 ± 0.009^Db^	6.34 ± 0.01^Da^
**TBARS (mg malonaldehyde/Kg.)**					
**RF (30%)**	0.38 ± 0.006^Ca^	0.46 ± 0.008^Bb^	0.65 ± 0.007^Cc^	0.79 ± 0.02^Ad^	1.28 ± 0.01^BCe^
**CF (20%)**	0.30 ± 0.01^Aa^	0.43 ± 0.01^Ab^	0.54 ± 0.01^Ac^	0.64 ± 0.008^Ad^	1.18 ± 0.02^Ae^
**BGF (10%)**	0.35 ± 0.01^Ba^	0.54 ± 0.007^Cb^	0.61 ±0.009 ^Bc^	0.73 ± 0.009^Bd^	1.26 ± 0.02^Be^
**PNF (10%)**	0.44 ± 0.006^Da^	0.56 ± 0.01^Db^	0.70 ± 0.010^Dc^	0.78 ± .008^Cd^	1.31 ± 0.11^Ce^

^*^Mean **±** SE with different superscripts in row (lower case alphabet) and column (Upper case alphabet) differ significantly (p < 0.05), n = 6 for each treatment, RC = Rice flour (control), CF = Corn flour, BGF = Black gram flour, PNF = peanut flour.

### pH

The effect of storage was obvious as the pH of fish curls followed a decreasing trend at progressive storage intervals. There was a significant decrease (p < 0.05) in pH of almost all variants except the curls made with 10 percent black gram in which the decrease was non-significant (p > 0.05) on day 7 and 14 and also on day 21 and 28. The decrease in pH might be attributed to the availability of more readily utilizable carbohydrate molecules by the microbes and thereby formation of lactic acid. It is an established fact that a decrease in pH is usually attributed to the metabolic activity of bacteria (Jay 1996). The decrease in pH in meat products also depended on the presence of fermentable carbohydrates (Borch et al. 1996). Singh et al. (2011) reported gradual decrease in the pH of chicken snacks stored in laminated pouches at ambient temperature. Between the treatments, the curls made with the incorporation of 10 percent peanut flour had significantly (p < 0.05) higher pH at all intervals of storage. This could be possibly due to more basic nature of peanut flour than rest of the flours used in the formulation. Chang et al. (2010) reported a similar decrease in pH during storage in sausages treated with carrot and onion. García et al. (2002) also observed a similar decrease in pH of low fat dry fermented sausages prepared with cereals and fruit fibers. Incze (1992) reported that decrease in the pH values might be due to significant (P < 0.05) increase in microbial count during storage period producing lactic acid by breakdown of carbohydrates. Murguerza et al. (2002) also reported a progressive decrease in pH of dry fermented sausages.

### Thiobarbituric acid reacting substances value (mg malonaldehyde/Kg)

Thiobarbituric acid reacting substances (TBARS) value followed a significantly (p < 0.05) increasing trend from day 0 to 28 in case of both control and treated curls. TBARS value of all products increased significantly (p < 0.05) at all intervals of storage. The increase in TBARS values on storage might be attributed to oxygen permeability of packaging material (Brewer et al. 1992) that led to lipid oxidation. Ratanatriwong et al. (2011), Singh et al. (2011) and Park et al. (1993) reported gradual increase in the TBARS values in fish, beef and chicken snacks respectively stored at ambient temperature. Chidanandaiah et al. (2009), Modi et al. (2003), Kumar and Tanwar (2011), Sudheer et al. (2011), Bhat and Pathak (2011) and Bhat et al. (2010) also reported a similar increase in TBARS values upon storage of different meat products. Between the treatments the TBARS value of corn flour curls were significantly lower as compared to other treatments at all intervals of storage. This might be possibly due to low fat content of corn flour used in the preparation of curls. However, in the present study TBARS values were much lower than threshold value of 2 mg/kg (Greene and Cumuze 1982) even by day 28. Similar findings were reported by Bhat et al. (2013a) and Bhat et al. (2011) in chicken *seekh kababs* extended with different non-meat proteins. Similar results were observed by Das et al. (2013) in chicken nuggets containing fermented bamboo shoot and Banerjee et al. (2012) in goat meat nuggets containing broccoli powder extract.

### Microbiological characters

The mean values of various microbiological characteristics of aerobically packaged fish curls containing different flours are presented in Table 3.

**Table 3:** **Effect of ambient storage on the microbiological characteristics of aerobically packaged fish curls incorporated with rice, corn, black gram and peanut flour (Mean ± SE)** ^*^

Treatments	Storage period (days)				
	0	7	14	21	28
**Total plate count (log cfu/g)**					
**RF (30%)**	2.24 ± 0.05^e^	2.79 ± 0.31^d^	3.35 ± 0.04^c^	4.73 ± 0.04^b^	5.46 ± 0.04^a^
**CF (20%)**	2.21 ± 0.29^e^	2.50 ± 0.33^d^	3.42 ± 0.06^c^	4.58 ± 0.03^b^	5.36 ± 0.03^a^
**BGF (10%)**	2.30 ± 0.02^e^	2.77 ± 0.23^d^	3.43 ± 0.04^c^	4.70 ± 0.05^b^	5.51 ± 0.03^a^
**PNF (10%)**	2.29 ± 0.01^e^	2.89 ± 0.03^d^	3.47 ± 0.05^c^	4.66 ± 0.04^b^	5.34 ± 0.03^a^
**Coliform count (log cfu/g)**					
**RF (30%)**	ND	ND	ND	ND	2.56 ± 0.05
**CF (20%)**	ND	ND	ND	ND	2.45 ± 0.01
**BGF (10%)**	ND	ND	ND	ND	2.62 ± 0.04
**PNF (10%)**	ND	ND	ND	ND	2.62 ± 0.07
**Yeast and mould count (log cfu/g)**					
**RF (30%)**	ND	ND	ND	2.48 ± 0.01^Ba^	3.53 ± 0.01^Bb^
**CF (20%)**	ND	ND	ND	2.32 ± 0.02^Aa^	3.46 ± 0.01^Ab^
**BGF (10%)**	ND	ND	ND	2.70 ± 0.05^Ca^	3.78 ± 0.01^Db^
**PNF (10%)**	ND	ND	ND	2.56 ± 0.01^Ba^	3.63 ± 0.02^Cb^

*Mean **±** SE with different superscripts in a row (lower case alphabet) and column (Upper case alphabet) differ significantly (p < 0.05), n = 6 for each treatment, RC = Rice flour (control), CF = Corn flour, BGF = Black gram flour, PNF = peanut flour, ND = Not detected.

### Total plate count (log cfu/g)

Total plate count (TPC) followed a significantly (p < 0.05) increasing trend as the storage days progressed in control as well as treated curls. However, total plate count showed a non-significant (p > 0.05) difference among different treatments at all the intervals of storage. Singh et al. (2011) also reported an increase in TPC at each storage interval in meat snacks. Similar findings were reported by Kumar et al. (2007) in chicken meat patties who also reported an increase in total plate count at each storage interval both in control and treatment samples. This is also in agreement with the findings of Chidanandaiah et al. (2009), Kumar and Tanwar (2011), Bhat et al. (2010), Bath and Pathak (2011), Bhat et al. (2013a) and Bhat et al. (2013b) who also reported the similar results in meat patties, chicken nuggets, chevon *Harissa*, mutton *Harrisa*, chicken *seekh kababs* and chicken meat balls respectively.

The higher TPC observed in fish curls in the present study might be probably due to easy availability of carbohydrate-rich starch in the fortified snack product to favor microbial growth. However, in the present study TPC count of control and treatment curls did not exceed the permissible level of microbial standards (log 10^6^ cfu g^_1^ of sample) in cooked meat products as reported by Jay (1996) even on 28^th^ day of storage.

### Coliform count (log cfu/g)

The coliforms were not detected in all treatments and control up to 21^st^ day of storage. This could be due to the destruction of these bacteria during cooking at 180 ± 5°C, far above their thermal death point of 57°C; hygienic practices followed during the preparation and packaging of curls. However, the counts appeared in all treatments on day 28. The count in control and treatments were comparable to each other. Appearance of coliforms later onwards could be because of contamination. Singh et al. (2011) have reported similar findings in chicken snacks at ambient temperature. Similar results were reported by Kumar and Sharma (2004) in pork patties, Kandeepan et al. (2010) in buffalo meat *keema*, Bhat et al. (2010) in chevon *Harrisa*, Bhat and Pathak (2011) in mutton *Harrisa*, Bhat et al. (2013a) in chicken *seekh kababs* and Bhat et al. (2013b) in chicken meat balls who also reported zero count of coliform for the products heated to such a high temperature.

### Yeast and mould count (log cfu/g)

The yeast and mould counts were not detected up to 14^th^ day of storage. However they appeared on day 21 onwards and followed a significantly (p < 0.05) increasing trend in all the treatments. The detection of yeast and mold counts on day 21^st^ and 28^th^ possibly could be due to post processing contamination. Singh et al. (2011) reported that yeast and mold appeared during the last day of storage of chicken snacks due to the availability of nutrients in meat. Among the treatments the counts were significantly (p < 0.05) higher for snacks containing black gram flour both on 21^st^ and 28^th^ day of storage. Das et al. (2013) also reported similar results in chicken nuggets.

### Sensory parameters

**Figure 2:**
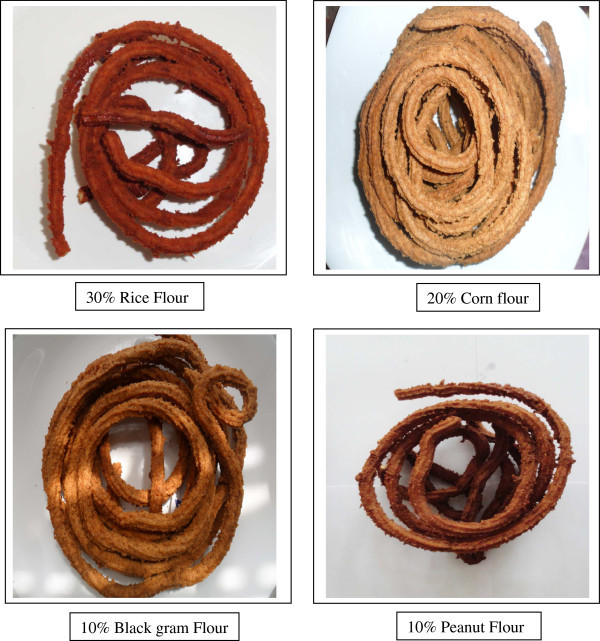
**Fish curls incorporated with optimum levels of different flours.**

Mean sensory scores of aerobically packaged fish curls containing different flours during ambient storage are presented in Table 4. Figure 2 shows the fish curls incorporated with optimum levels of different flours.

**Table 4:** **Effect of ambient storage on sensory attributes of aerobically packaged fish curls incorporated with rice, corn, black gram and peanut flour (Mean ± SE)** ^*^

Treatments	Storage period (days)				
	0	7	14	21	28
**Colour and appearance**					
**RF (30%)**	6.28 ± 0.11^Ac^	6.21 ± 0.11^Ac^	6.02 ± 0.08^Ac^	5.78 ± 0.12 ^Ab^	5.26 ± 0.17^a^
**CF (20%)**	6.88 ± 0.06^Bc^	6.78 ± 0.08^Bc^	6.57 ± 0.10^Ac^	6.28 ± 0.12^Ab^	5.21 ± 0.16^a^
**BGF (10%)**	7.45 ± 0.07^Cc^	7.33 ± 0.07^Cc^	6.84 ± 0.10^Bc^	6.73 ± 0.13^Bb^	5.30 ± 0.20^a^
**PNF (10%)**	7.50 ± 0.09^Cc^	7.42 ± 0.09^Cc^	7.23 ± 0.05 ^Bc^	6.83 ± 0.07^Bb^	5.45 ± 0.10_a_
**Flavour**					
**RF (30%)**	6.42 ± 0.11^Ac^	6.26 ± 0.13^Ac^	6.19 ± 0.09^Ac^	5.57 ± 0.11^Ab^	4.57 ± 0.13^a^
**CF (20%)**	6.78 ± 0.11^Bc^	6.54 ± 0.10^Ac^	6.45 ± 0.09^Ac^	5.61 ± 0.10^Ab^	4.76 ± 0.17^a^
**BGF (10%)**	7.19 ± 0.08^Cd^	6.97 ± 0.10^Ccd^	6.78 ± 0.11^Bc^	5.52 ± 0.11^Ab^	4.30 ± 0.22^a^
**PNF (10%)**	7.23 ± 0.06^Cc^	7.11 ± 0.06_Cc_	6.90 ± 0.07^Bc^	5.92 ± 0.09^Bb^	4.80 ± 0.17^a^
**Crispiness**					
**RF (30%)**	6.26 ± 0.13^Ac^	6.19 ± 0.12^Ac^	5.95 ± 0.09_Ac_	5.47 ± 0.012^b^	4.59 ± 0.11^a^
**CF (20%)**	7.19 ± 0.08^Bc^	7.09 ± 0.09^Bc^	6.51 ± 0.10_Bb_	5.92 ± 0.14^b^	4.66 ± 0.15^a^
**BGF (10%)**	7.04 ± 0.04_Bd_	6.95 ± 0.05^Bd^	6.58 ± 0.11_Ac_	5.97 ± 0.90^b^	4.76 ± 0.15^a^
**PNF (10%)**	7.16 ± 0.09_Bd_	7.09 ± 0.05^Bd^	6.76 ± 0.08_Bc_	6.07 ± 0.11^b^	4.88 ± 0.10^a^
**Texture**					
**RF (30%)**	6.83 ± 0.12^BCc^	6.64 ± 0.12^Ac^	6.42 ± 0.11^Ab^	6.28 ± 0.11^b^	5.64 ± 0.11^a^
**CF (20%)**	6.47 ± 0.09^Ac^	6.40 ± 0.08^Abc^	6.30 ± 0.08^Abc^	6.11 ± 0.11^b^	5.76 ± 0.11^a^
**BGF (10%)**	7.09 ± 0.09^Cc^	7.02 ± 0.08^Bc^	6.78 ± 0.10^Bc^	6.30 ± 0.15^b^	5.64 ± 0.10^a^
**PNF (10%)**	6.76 ± 0.10^ABc^	6.69 ± 0.10^Ac^	6.54 ± 0.10^ABbc^	6.33 ± 0.12^b^	5.97 ± 0.11^a^
**Overall acceptability**					
**RF (30%)**	6.35 ± 0.10^Ac^	6.26 ± 0.11^Ac^	6.07 ± 0.10 ^Ac^	5.69 ± 0.13^b^	4.28 ± 0.14^a^
**CF (20%)**	7.11 ± 0.08^Bc^	6.97 ± 0.12^Bc^	6.64 ± 0.11^Bc^	5.92 ± 0.07^b^	4.59 ± 0.19^a^
**BGF (10%)**	7.26 ± 0.07^BCd^	7.14 ± 0.08^Bc^	6.73 ± 0.09^Bc^	5.76 ± 0.16 ^b^	4.23 ± 0.16^a^
**PNF (10%)**	7.45 ± 0.09^Cc^	7.26 ± 0.08^Bc^	6.88 ± 0.08^Cc^	5.95 ± 0.11^b^	4.71 ± 0.12^a^

*Mean **±** SE with different superscripts in a row (lower case alphabet) and column (Upper case alphabet) differ significantly (p < 0.05), n = 6 for each treatment, RC = Rice flour (control), CF = Corn flour, BGF = Black gram flour, PNF = peanut flour.

### Colour and appearance

The colour and appearance scores followed a linear decreasing trend as the storage days progressed. This decrease was significantly (p < 0.05) highest on 21^st^ and 28^th^ day of storage. The scores showed a non-significant (p > 0.05) decrease up to day 14^th^ in all the products. A similar decrease in appearance and colour scores of chicken and fish snacks with increase in storage period was also reported by Singh et al. (2011) and Ratanatriwong et al. (2011) respectively. Similar findings were also reported by Kumar and Sharma (2004) in chicken patties, Kilinc (2009) in anchovy patties, Chidanandaiah et al. (2009) in buffalo patties, Bhat et al. (2010) in chevon *Harrisa*, Bhat et al. (2013a) in chicken *seekh kababs* and Bhat et al. (2013b) in chicken meat balls respectively.

Among the treatments the colour and appearance scores were comparable (p > 0.05) on 28^th^ day of storage. However on day 0, 7, 14 and 21 the scores among the treatments were significantly (p < 0.05) higher in the curls incorporated with black gram and peanut flour in the formulation as compared to control and corn flour incorporated curls. This could be possibly attributed to the suitable golden colour imparting property of these flours.

### Flavour

Flavour of the products decreased gradually (p > 0.05) as the days of storage progressed and on 28^th^ day of storage the score was significantly (p < 0.05) lowest. The reduction in flavour could be attributed to the increased lipid oxidation, liberation of fatty acids and increased microbial load (Sahoo and Anjaneyulu 1997). A gradual decline of flavour might also be due to the expected loss of volatile flavour components from spices and condiments on storage of meat products. The progressive decrease in flavour could be correlated to increase in TBARS value of meat products (Tarladgis et al. 1960) stored under aerobic conditions. Decline in flavour scores of meat snacks during storage was also reported by Singh et al. (2011). The decrease in flavour and meat flavour scores with the advancement of the storage period might also be due to dilution in meaty flavour. Similar reports have been published by Padda et al. (1989), Kumar and Sharma (2004), Bhat and Pathak (2009), Bhat et al. (2013a) and Bhat et al. (2013b) for various meat products. However, among the treatments the flavour score showed a non-significant (p > 0.05) difference on 28^th^ day of storage. The flavour score among the treatments was significantly (p < 0.05) higher in the curls incorporated with black gram and peanut flour in the formulation as compared to control and corn flour incorporated curls on 0, 7 and 14^th^ day of storage.

### Crispiness

Crispiness decreased gradually as the days of storage progressed and on 28^th^ day of storage the score was significantly (p < 0.05) lowest. The score was non-significant up to 7^th^ day of storage. Crispiness scores might have decreased because of moisture absorption with the increase in storage days. However, among the different treatments the scores showed a significant (p < 0.05) difference on 0, 7 and 14^th^ day of storage, whereas on 21^st^ and 28^th^ day of storage the scores were comparable (p > 0.05).

### Texture

Texture of the products decreased linearly as the storage progressed and the scores were comparable (p > 0.05) up to 14^th^ day of storage in all the treatments. The decrease in scores was significant (p < 0.05) 21^th^ day onwards. However, among the different treatments the scores showed a non-significant (p > 0.05) difference on 21^st^ and 28^th^ day of storage. Similar observations have also been reported by Kumar and Sharma (2004), Bhat and Pathak (2009), Bhat et al. (2013a) and Bhat et al. (2013b) for various meat products. Kalra et al. (1987) also observed slight decrease in the texture scores of snacks packaged in low density polyethylene (LDPE) pouches.

### Overall acceptability

Overall acceptability of control as well as flour incorporated curls decreased linearly as the storage days progressed. The scores showed a non-significant decrease up to 14^th^ day of the storage in almost all the products. However, among the treatments the scores showed a non-significant (p > 0.05) difference on 21^st^ and 28^th^ day of storage. The scores among the treatments showed a significant (p < 0.05) difference on 0, 7 and 14^th^ day of storage. The overall acceptability scores between the treatments were significantly (p < 0.05) higher in peanut flour incorporated curls on day 0 and 14^th^ of the storage as compared to rest of the treatments. The decline in overall acceptability scores could be attributed to changes in scores of colour and appearance, flavour, texture and other sensory attributes. Similar findings have also been reported by Kumar and Sharma (2004), Bhat and Pathak (2009), Bhat et al. (2013a) and Bhat et al. (2013b) for various meat products.

## Conclusions

Fish curls of very good palatability could be prepared by incorporating 20 percent corn flour or 10 percent black gram flour or 10 percent peanut flour in formulation substituting rice flour. Although, flours improved the sensory attributes, both control as well as flour incorporated fish curls could be conveniently packed in LDPE pouches for a period of 21 days in ambient conditions without any marked loss of physicochemical, microbial and sensory quality. Thus, the present study showed successful utilization of different flours and fish in the preparation of curls.
